# Management of cardiovascular risk in patients with multiple myeloma

**DOI:** 10.1038/s41408-019-0183-y

**Published:** 2019-02-26

**Authors:** Chris Plummer, Christoph Driessen, Zsolt Szabo, María-Victoria Mateos

**Affiliations:** 1 Department of Cardiology, Freeman Hospital, Freeman Road, Newcastle upon Tyne, NE7 7DN UK; 2 Department of Oncology and Hematology, Kantonsspital St Gallen, Rorschacher Strasse 95, CH-9007 St Gallen, Switzerland; 3 Amgen (Europe) GmbH, Suurstoffi 22, 6343 Rotkreuz, Switzerland; 4 Hematology Service, University Hospital Salamanca, Casa del Bedel, Cardenal Pla y Deniel, 22, Planta Baja, Salamanca, 37008 Spain

## Abstract

Multiple myeloma (MM) is a plasma cell malignancy that accounts for 10% of hematological cancers. It predominantly affects elderly people; median age at diagnosis is 70 years. Consequently, many patients with MM have cardiovascular comorbidities or risk factors. MM can cause cardiac comorbidities such as cardiomyopathy and heart failure caused by cardiac amyloidosis and/or anemia. Some of the treatments used in MM can also affect cardiovascular health. Advances in pharmacotherapy for MM, such as the introduction of immunomodulators, proteasome inhibitors, histone deacetylase inhibitors, and monoclonal antibodies, have dramatically improved progression-free survival and life expectancy, but new agent classes are associated with adverse events that were not previously observed on a regular basis, including cardiovascular events. However, with careful risk assessment, monitoring, and prophylactic therapy, many of these cardiovascular complications can be managed or treated successfully. Most routine cardiovascular surveillance is undertaken by the treating hemato-oncologist, but a multidisciplinary approach involving cardiologists may help to optimize patient outcomes. In this review, we survey the cardiac complications commonly reported in patients with MM, discuss how they can be prevented and managed, and summarize the role cardiologists can play in delivering the best possible outcomes for patients with MM and cardiovascular comorbidities.

## Introduction

Multiple myeloma (MM) is a malignancy of plasma cells, accounting for ~ 10% of all hematological cancers^1,2^. Patients with MM are often elderly; estimates suggest that the median age at diagnosis is ~ 70 years^3^. Consequently, many of these patients have cardiovascular risk factors or comorbidities at diagnosis^4^. In addition, the disease itself can have direct and indirect detrimental effects on cardiac function.

During the course of their disease, patients with MM are usually exposed to several treatments, often in combination, that may each increase the risk of cardiovascular adverse events (AEs). As a consequence, assessing cardiovascular risk and controlling cardiovascular complications are becoming integral to the routine management of patients with MM. Advances in treatment have increased life expectancy^5^, placing a greater emphasis on minimizing long-term toxicity. A multidisciplinary approach, with the input of cardiologists, may improve outcomes in patients with MM who have cardiovascular comorbidities.

Here, we summarize the underlying cardiovascular risks in patients with MM and review the nature of cardiovascular AEs that can occur during treatment. We also describe how these risks can be minimized and how complications can be treated effectively in collaboration with the cardiologist.

Due in part to the rapidly evolving treatment landscape, up-to-date real-world evidence on safety in patients with MM is limited. Furthermore, MM is a heterogeneous disease with considerable variation in presentation and comorbidities, making comparisons between clinical trials in patients with MM particularly difficult. Therefore, this review will focus predominantly on data from summaries of product characteristics (SmPCs) and individual phase 3 clinical trials. The classification of selected cardiovascular AEs according to the Common Terminology Criteria for Adverse Events (version 5) is available in Supplementary Table 1^6^, whereas Supplementary Table 2 presents a summary of cardiovascular AEs reported in key phase 3 trials involving agents used in the treatment of patients with relapsed and/or refractory MM (RRMM)^7–33^. Real-world patient populations differ from those in clinical trials. The former are likely to be older and have more comorbidities, and this should be taken into account when considering the rates of cardiovascular AEs reported in this review. In contrast, patients in clinical trials are likely to have mandated levels of renal, hepatic, and cardiac function. Many of the new agents were approved in recent years, such that long-term, real-world, safety data on these emerging treatments are not yet available. The need for strong clinical collaborations between hematologists and cardiovascular specialists in routine clinical practice is crucial.

## Pathophysiology of myeloma, and baseline and disease-related cardiovascular complications

Rapidly proliferating malignant B-cells secrete large quantities of immunoglobulins or immunoglobulin fragments into the bloodstream, which can collect in organs including the heart, liver, and kidneys^34^. The accumulation of amyloid light-chain (AL) immunoglobulin is estimated to lead to clinical amyloidosis in 12–15% of patients with MM during the course of their disease, and up to 30% of those with MM have subclinical amyloid deposits^34^. Cardiac involvement is estimated to be present in 50% of all AL amyloidosis cases^35,36^. MM is associated with a specific set of clinical manifestations often referred to as the CRAB features (elevated calcium levels, renal insufficiency, anemia, and bone lesions)^37^, some of which can also increase the risk of cardiovascular comorbidities. For example, hypercalcemia is linked to the development of arrhythmias^38^, renal insufficiency has been shown to increase the risk of cardiovascular disease by two to four times^39^, and a study on atherosclerosis risk in the community suggested that iron deficiency (anemia) is an independent risk factor affecting cardiovascular disease outcomes in patients aged 45–64 years^40^. Anemia can also lead to arrhythmia, cardiomyopathy, and high-output heart failure^41,42^.

Given the combination of disease-related factors discussed above and the age of individuals with MM, it is not surprising that many have existing cardiovascular morbidity. Indeed, MM is primarily a disease of the elderly, an age group with a high burden of cardiovascular complications^3^. In the United States in 2005, prevalence of cardiovascular disease (including hypertension, coronary heart disease, heart failure, and stroke) was estimated to be ~40% in patients aged 40–59 years, increasing to 70–75% in those aged 60–79 years and to 79–86% for those aged 80 years and older^43^. A study from the USA found that 63% of individuals newly diagnosed with MM had a history of cardiac events^4^; and pooled European data from six randomized trials showed that 69% of patients with MM had cardiovascular comorbidities at diagnosis^44^.

## Overview of the treatment landscape

**Fig. 1:**
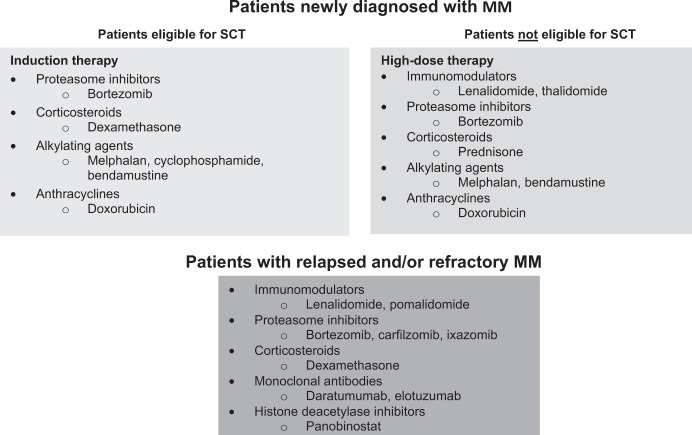
Summary of agents approved for the treatment of patients with MM in Europe^8,49–51,55,56,67,69,73,80–82,88^. MM, multiple myeloma; SCT, stem-cell transplantation

Patients with MM, especially those with RRMM, are likely to be exposed to a number of treatment modalities and pharmacotherapeutic agents during the course of their disease (Fig. 1)^45,46^. The specific sequence of regimens used will depend on factors such as age, fitness, line of therapy, and response to previous treatments. Eight classes of agents are commonly used in the treatment of MM: anthracyclines, proteasome inhibitors, immunomodulators, alkylating agents, corticosteroids, monoclonal antibodies, histone deacetylase inhibitors, and tubulin polymerization inhibitors^7,45^. In addition to treatment with chemical and biological agents, many patients with MM will undergo stem-cell transplantation (SCT); it should be noted, however, that SCT is not generally considered to be appropriate for elderly patients or those with serious comorbidities (including those with cardiac comorbidities)^46^. Palliative radiotherapy is also widely used in patients with MM and evidence suggests that almost one-third of patients will receive radiotherapy at some point during the course of the disease^47^.

### Anthracyclines (doxorubicin)

Doxorubicin is widely used in the treatment of patients with MM. Before the development of novel therapies, doxorubicin was regularly employed in the first-line setting, but it is now predominantly used in later treatment lines^48^. Typically, doxorubicin is administered in a polyethylene glycol (PEG)-ylated liposomal formulation at a dose of 30 mg/m^2^ on day 4 of each 21-day cycle in combination with the proteasome inhibitor bortezomib for up to a maximum of eight cycles (cumulative dose of 240 mg/m^2^)^49,50^. Cardiotoxicity is a well-known risk associated with anthracycline treatment. Doxorubicin hydrochloride (in a non-PEGylated liposomal formulation) has a recommended lifetime cumulative dose limit of 450–550 mg/m^2^, but cardiac toxicity can occur at much lower doses^51^. As a result, intravenous non-PEGylated liposomal doxorubicin is contraindicated in patients with severe arrhythmia, previous myocardial infarction, heart failure, or acute inflammatory heart disease^51^. The exact mechanisms through which anthracyclines cause cardiac damage are not fully understood, but it is widely accepted that many pathways are involved^52^. The anthracycline-induced generation of reactive oxygen species (ROS) is believed to be a key factor because exposure to ROS can damage several cell components, ultimately leading to cell death^52,53^. Other postulated mechanisms include prevention of DNA and RNA synthesis through anthracycline intercalation and activation of the mitogen-activated protein kinase/extracellular signal-related kinase pathway^54^.

### Proteasome inhibitors

Three proteasome inhibitors have been granted European approval for use in patients with MM: bortezomib, initially approved for patients with RRMM in 2004 and later approved for the treatment of patients newly diagnosed with MM^50^; carfilzomib, approved for use in patients with RRMM in 2015^55^; and ixazomib, approved in 2016 for use in patients with MM who have received at least one previous treatment^56^. Bortezomib, carfilzomib, and ixazomib are also approved for use in MM in the USA^57–59^.

The ubiquitin–proteasome system is responsible for the degradation of intracellular proteins and the maintenance of cellular protein homeostasis^2^. Proteasome inhibition leads to an intracellular accumulation of aggregated proteins that is disproportionally toxic to MM cells^60,61^. However, the heart is a very metabolically active organ with a resting metabolic rate that is more than twice as high as that of the liver^62^ and cardiac myocytes are also sensitive to proteasome inhibition^63^. Consequently, cardiac dysfunction may occur if protein homeostasis is not maintained, potentially leading to heart failure^64^. Furthermore, evidence suggests that the ubiquitin–proteasome system is dysfunctional during myocardial ischemia. Experiments in transgenic mice have demonstrated that proteasome inhibition in cardiac myocytes contributed to the development of heart failure during systolic overload^65^, and it is postulated that the degree of cardiac injury may be correlated to the level of proteasome inhibition^64^. Cardiac AEs have been reported following treatment with each of the available proteasome inhibitors, but are most commonly associated with carfilzomib treatment^13,50,55,66^.

### Immunomodulators

Immunomodulators are widely used in the treatment of patients newly diagnosed with MM and those with RRMM^45,46^. The first-generation immunomodulators, lenalidomide and thalidomide, received European approval for use in patients with MM in 2007 and 2008, respectively^67,68^, and pomalidomide, a second-generation immunomodulator, received marketing authorization for use in patients with RRMM in 2015^69^. Treatment with these agents, especially when combined with corticosteroids and other chemotherapies, is associated with an increased risk of venous thromboembolism (VTE). The exact mechanisms underlying immunomodulator-induced thromboembolism are not fully understood. It is postulated, however, that such agents can alter the equilibrium between procoagulant and anticoagulant proteins on the surface of endothelial cells^64^. The full mode of action through which immunomodulators exert an antineoplastic effect is yet to be elucidated^70^, but it is believed that inhibition of angiogenesis is a contributory factor^67,69^. Other agents that inhibit angiogenesis, such as sorafenib and bevacizumab, are associated with cardiovascular AEs including hypertension and congestive heart failure^71,72^.

### Monoclonal antibodies

Monoclonal antibodies have recently entered the MM treatment landscape for patients with relapsed or refractory disease. Elotuzumab, a monoclonal antibody that targets signaling lymphocytic activation molecule F7, is approved in combination with lenalidomide and low-dose dexamethasone for the treatment of patients with RRMM^8^. Daratumumab, a monoclonal antibody targeting CD38, is approved in Europe as monotherapy or in combination with either lenalidomide or bortezomib, plus dexamethasone for patients with RRMM^73^.

CD38 is a multifunctional protein that has enzymatic properties in addition to receptor-mediated adhesion and signaling activity^73^. It catalyzes the metabolism of cyclic adenosine diphosphate-ribose and nicotinic acid adenine dinucleotide phosphate^74^. CD38 is expressed on the surface of myeloma cells, but it is also expressed on the surface of many other cells, including erythrocytes and cardiomyocytes^46,73,75^. An in vitro study has shown that daratumumab induces the loss of CD38 from erythrocytes, but no hemolysis was observed^75^. Another study has shown that the adhesion of lymphocytes to endothelial cells is inhibited by the presence of a monoclonal antibody that binds to CD38^76^.

### Corticosteroids

Dexamethasone and prednisone are widely used in MM treatment regimens. Corticosteroid use is not directly associated with serious cardiovascular toxicity, although prolonged use is associated with fluid retention and the development of type 2 diabetes mellitus and hypertension, both of which can increase the risk of cardiovascular events^77,78^. The effect that dexamethasone can have on the incidence of cardiovascular events was highlighted during a phase 3 trial of lenalidomide combined with two different doses of dexamethasone in patients newly diagnosed with MM^79^. The incidence of deep vein thrombosis or pulmonary embolism at grade 3 or above was significantly higher in patients receiving lenalidomide with high-dose dexamethasone (40 mg on days 1–4, 9–12, and 17–20 of each 28-day cycle) than in those receiving lenalidomide with low-dose dexamethasone (40 mg on days 1, 8, 5, and 22 of each 28-day cycle) (26% and 12%, respectively)^79^. Similar incidences of thromboembolic events were observed in patients with RRMM who received lenalidomide and high-dose dexamethasone in clinical studies. The rates of grade 3 or 4 VTE in patients who received this regimen in two phase 3 trials were 11.4% and 14.7%, respectively^9,10^. In the same populations, 4.5% and 3.4% of patients, respectively, experienced grade 3 or 4 pulmonary embolism, and 4.0% and 11.9% of patients, respectively, had grade 3 or 4 deep vein thrombosis^9,10^. One on-study death was attributed to pulmonary embolism^9^.

### Alkylating agents

Melphalan and bendamustine are approved for the first-line treatment of patients newly diagnosed with MM who are not suitable for SCT^80,81^. Cyclophosphamide, in combination with bortezomib and dexamethasone, is used in the first-line setting in both transplant-eligible patients and those who are not suitable for SCT^82^. Cardiotoxicity has been reported with cyclophosphamide treatment and has led to severe, occasionally fatal, congestive heart failure. The risk of cardiotoxicity increases with higher drug doses as well as with the presence of underlying risk factors, such as advanced age^82^. Bendamustine has been associated with myocardial infarction and cardiac failure, and is also associated with arrhythmia^81^. In addition, treatment with autologous SCT and high-dose melphalan may lead to atrial and supraventricular arrhythmia^83–85^.

### Histone deacetylase inhibitors

Histone deacetylase 6 is an enzyme that facilitates several steps of the aggresome pathway, which has a pivotal role in intracellular protein homeostasis^86^. Agents that inhibit this enzyme (and other histone deacetylases), such as panobinostat, increase the quantity of cytotoxic aggregated proteins inside MM cells, leading to cell death^87^. Panobinostat, in combination with bortezomib and low-dose dexamethasone, was approved for the treatment of patients with RRMM in Europe in 2015^88^.

### Radiotherapy

In a study of 149 patients who received palliative radiotherapy, the thoracic spine was the most common site of irradiation, and the most frequency used dosing schedule was 30 Gy given in 10 fractions^47^. Data on cardiac events associated with radiotherapy in patients with MM are limited. However, evidence from patients with breast cancer (who typically receive a dose of 40 Gy in 15 fractions in the adjuvant setting) suggested that the relative risk of fatal cardiovascular events following left-side radiation was 1–2.2 compared with patients who did not receive radiotherapy^89,90^. Risk factors for radiation-related cardiovascular damage include: total dose ( > 30–35 Gy), dose per fraction ( > 2 Gy), age at exposure, and presence of baseline cardiovascular risk factors (e.g., hypertension, diabetes, obesity, or smoking)^89^.

## Managing cardiovascular risk in patients with MM

### Assessing cardiovascular risk at baseline

As the treatment of patients with MM has evolved, so have the understanding and management of AEs associated with each new treatment. Over time, strategies have been developed to manage hematological toxicities associated with high-dose chemotherapy^91^, VTE associated with immunomodulators^92^, and peripheral neuropathy related to bortezomib^93^. In the absence of anthracyclines, treatment-related cardiac AEs are a relatively new challenge for the MM community; therefore, no disease-specific guidelines have been published on this topic. However, guidance on the management of cardiac events associated with chemotherapy agents and radiotherapy has been published by the European Society for Medical Oncology (ESMO) and the European Society of Cardiology (ESC)^64,94,95^. These guidelines recommend that before therapy with anthracyclines or any other treatment with potential cardiotoxicity is initiated, all patients should be assessed for cardiovascular risk factors such as obesity, smoking, diabetes mellitus, hypertension, hypercholesterolemia, personal and family history of cardiovascular disease, and previous treatment with 5-hydroxytryptamine-2-β agonists^95^. The guidelines also suggest that all patients should be given evidence-based advice to reduce their cardiovascular risk^64^. Consideration of the mechanism underlying cardiac AEs is relevant; for example, in its review of the risk–benefit profile of carfilzomib, the European Medicines Agency proposed that patients with elevated cardiovascular risk need not be prevented from receiving the drug (as long as a comprehensive assessment is conducted before treatment commences), on the basis that cardiac effects associated with this agent are likely pharmacological^96^.

Baseline cardiac function can be assessed by clinical examination, electrocardiography (ECG), echocardiography, and multigated acquisition scans^89^. Typically, echocardiography is used to measure left ventricular ejection fraction (LVEF) and to assess cardiac structure and valve function. Measurement of baseline brain natriuretic peptide (BNP) and its precursor, N-terminal prohormone BNP (NT-proBNP), can also be used to detect cardiac dysfunction, but its use is limited by low specificity in patients with MM^89^. Local guidelines recommend that blood pressure is assessed on day 1 of each cycle of treatment (both lying down and standing up for the first cycle) for many of the commonly used antimyeloma regimens^97–99^.

Patients treated with carfilzomib are at a greater risk of cardiac failure if they are aged 75 years or over and may have higher risk of cardiac AEs, if they have any of the following risk factors: heart failure (with or without reduced LVEF), coronary artery disease, valvular heart disease, cardiomyopathy (hypertrophic, dilated, or restrictive), uncontrolled cardiac arrhythmia, or pre-treatment with anthracyclines. Patients with comorbidities (including hypertension, peripheral vascular disease, diabetes, or hypercholesterolemia), who smoke, who are obese, or who have sedentary habits can also be at greater risk of cardiac AEs^100^. It is likely these risk factors may also increase the risk of cardiac AEs in patients treated with other MM regimens.

### Monitoring patients during therapy

During treatment, patients and physicians should remain vigilant for cardiovascular signs or symptoms. Reports of dyspnea, chest pain, edema, or fatigue should be investigated to ascertain whether they are being caused by cardiac dysfunction. Dyspnea, for example, can also be caused by other conditions such as asthma, respiratory infection, or chronic obstructive pulmonary disease^101^. Monitoring of cardiac function during treatment using echocardiography and ECG is also advised in some regimens, although specific evidence-based protocols are lacking. A significant decline in LVEF ( ≥ 10% reduction to a value below the lower limit of normal ( ≤ 55%)) should prompt consideration of starting evidence-based treatment for left ventricular dysfunction using agents such as angiotensin-converting enzyme (ACE) inhibitors and β-blockers^64^. The decision whether or not to continue cancer treatment should be made on an individual basis, taking into account the relative risks of the patient’s MM and overall cardiovascular status, as well as the likely effects of the implicated drug^89^. In the case of carfilzomib treatment, consensus guidance from the European Myeloma Network (EMN) on AEs associated with novel agents in MM recommends that if a patient suffers a grade 3 or above cardiac AE, carfilzomib should be withheld, and fluid administration should be ceased. If appropriate, carfilzomib therapy may be restarted at one level dose reduction based on a risk–benefit assessment. Notably, data from a cardiac substudy of the ENDEAVOR trial suggest that reduction in LVEF with carfilzomib or bortezomib is mostly reversible^102^. When therapy is resumed, follow-up echocardiograms should be considered^100^. However, it should be noted that one study found that the utility of echocardiography as a tool to identify patients at risk of reduced LVEF and right ventricular ejection fraction was limited in patients receiving carfilzomib^103^.

### Venous thromboembolism

The incidence of VTE in patients with MM is estimated to be between 3% and 10%^11,104^, and baseline, disease, and treatment-related factors are all influential.

**Fig. 2:**
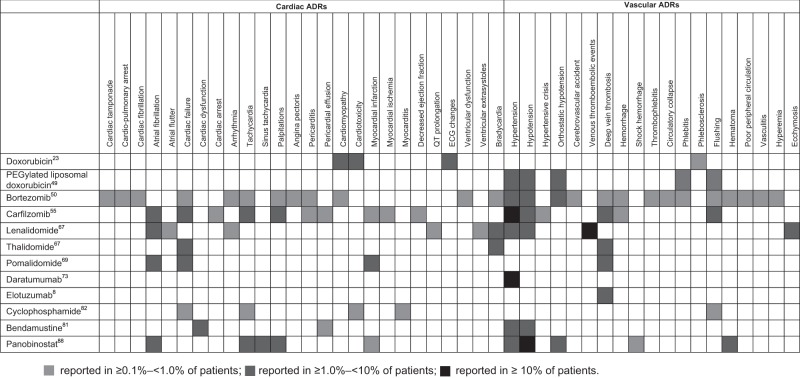
Summary of cardiovascular adverse drug reactions associated with agents used to treat MM in Europe and occurring in at least 1 in 1000 patients (all AEs listed with frequency information in the relevant European SmPCs are depicted). ADR, adverse drug reaction; ECG, electrocardiography; MM, multiple myeloma; PEG, polyethylene glycol; SmPC, summary of product characteristics

Treatment with immunomodulators is known to increase the risk of VTE. Pooled data from European SmPCs for these agents showed that grade 3 or 4 VTE (as defined by the common terminology for adverse events criteria) was classed as a very common adverse drug reaction (ADR) (occurring in > 10% of patients) for lenalidomide^67^, and deep vein thrombosis was classed as common (1.0–9.9% of patients) for thalidomide and pomalidomide (Fig. 2)^67,69^.

Management of VTE risk should also be taken into consideration when prescribing proteasome inhibitors, especially carfilzomib. Pooled data from multiple trials and postmarketing studies published in European SmPCs showed that VTEs of any grade were common in patients treated with carfilzomib^55^ and uncommon in those who received bortezomib^50^. The incidences of grade 3 or higher pulmonary embolism and deep vein thrombosis in patients treated with carfilzomib-containing regimens during phase 3 trials were 1.7–3.1 and 0.9–1.8%, respectively (Supplementary Table 2)^16–18^. The overall risk of VTEs in these trials was higher for carfilzomib-containing regimens than for carfilzomib-free regimens, regardless of whether they contained an immunomodulator^17,18^. Although direct comparisons cannot be made across trials owing to potential differences in patient populations, the incidence of grade 3 or higher VTEs was 5.6% in the carfilzomib arm of ASPIRE and 3.5% in the carfilzomib arm of ENDEAVOR; this suggests that rates of VTE may be higher when carfilzomib is used in combination with lenalidomide and dexamethasone than when combined with dexamethasone only^17,18,55^. The rates of grade 3 or 4 thromboembolic events in patients with MM who received bortezomib during phase 3 clinical trials were in the range 1–6% (Supplementary Table 2)^12,19–22^. In the phase 3 TOURMALINE-MM1 study, grade 3 or 4 thromboembolism occurred in 3.0% patients treated with oral ixazomib plus lenalidomide and dexamethasone, compared with 3.3% receiving lenalidomide plus dexamethasone (Supplementary Table 2)^13^.

VTE has also been observed in patients treated with the monoclonal antibody elotuzumab. In the phase 3 ELOQUENT-2 trial, 6.3% of patients with RRMM treated with a combination of elotuzumab, lenalidomide, and low-dose dexamethasone had grade 3 or 4 deep vein thrombosis (Supplementary Table 2)^33^.

Owing to the increased risk of VTE, additional monitoring is recommended for patients receiving lenalidomide, pomalidomide, or thalidomide^67,69^. The International Myeloma Working Group (IMWG) and the EMN have published guidelines on the management of VTE in patients with MM^92,100^. The recommendations take into account the number of risk factors present, which include age, body mass index, inherited thrombophilic abnormalities, central venous catheterization or pacemaker, previous superficial vein thrombosis, pregnancy/puerperium, drug use, recent surgery, trauma, hospitalization, nursing home confinement, malignant neoplasm, and neurological disease. Prophylactic aspirin is recommended for patients with zero or one risk factor, and low-molecular-weight heparin (LMWH) or full-dose warfarin should be given to those with two or more risk factors or those treated with immunomodulatory agents, high-dose dexamethasone, doxorubicin or multi-agent chemotherapy for at least the first 5 months of antimyeloma therapy^92,100^.

The IMWG advises that patients with MM who are treated with immunomodulators should stay vigilant for common signs or symptoms of deep vein thrombosis such as redness of the skin, pain in the extremities or chest, dyspnea, and rapid heartbeat^92^. In the event of a thromboembolic event during treatment, LMWH should be used as the first line of treatment^92^. Immunomodulator therapy should be discontinued, but can be resumed once full anticoagulation has been established^92^.

Carfilzomib-specific advice is available for the management of VTE risk^55^. Patients who have risk factors for thromboembolism should be monitored closely, and action should be taken to minimize modifiable risk factors (e.g., smoking cessation and antihypertensive therapy). The concomitant use of other treatments that can increase the risk of thromboembolism, such as erthyropoietic agents and hormone-replacement therapy, should also be considered carefully. Patients and clinicians should remain vigilant for the signs and symptoms of thromboembolism, and thromboprophylaxis should be considered based on an assessment of the patient’s underlying risks and clinical status^55^. Although this advice specifically relates to carfilzomib, these considerations could be useful for the management of all patients with MM including those receiving alternative therapies.

### Congestive heart failure

Many patients with MM will have cardiac conditions and risk factors at diagnosis. In a US study of 22,076 patients newly diagnosed with MM, 63% of individuals had a history of cardiac comorbidities, the most common being arrhythmias (14%), ischemic heart disease (14%), and congestive heart failure (8%)^4^. Of more concern, the study also showed that over 70% of patients with MM developed a cardiac complication during treatment (72% with newly diagnosed MM and 71% with relapsed MM). While on treatment, 24% of newly diagnosed patients and 29% of patients with relapsed MM developed an arrhythmia^4^.

Several drug classes used in the treatment of MM are known to increase the risk of cardiac events. Doxorubicin-related cardiac events can be acute or delayed. Acute events during doxorubicin treatment include sinus tachycardia and ECG abnormalities; left ventricular dysfunction usually develops after the completion of treatment^51^. The European SmPC for doxorubicin (in the non-PEGylated liposomal formulation) lists cardiotoxicity as a common ADR, and the probability of developing congestive heart failure during or following doxorubicin treatment is estimated to be 1–2% at a cumulative dose of 300 mg/m^2^. The risk increases with dose up to the maximum lifetime recommended cumulative limit of 450–550 mg/m^2^ and is highly dependent on the duration of follow-up because anthracycline-related heart failure can present decades after exposure^51^. PEGylated liposomal formulations of doxorubicin are associated with lower rates of cardiotoxicity than the non-PEGylated equivalent^49^. In a phase 3 clinical trial, the incidence of cardiac events of grade 3 or above was 2% in patients treated with PEGylated liposomal doxorubicin and bortezomib^20^.

European SmPCs list grade 3 or 4 cardiac failure as a common ADR for lenalidomide^67^ and pomalidomide^69^ and all-grade cardiac failure as a common ADR related to treatment with thalidomide^67^. In phase 3 trials, grade 3 or 4 cardiac failure was reported in 1.7–1.8% of patients treated with lenalidomide and low-dose dexamethasone^13,17^. Induction therapy with thalidomide in combination with bortezomib and prednisone led to grade 3 or above cardiac events in 8% of patients compared with 0% of patients who received a combination of melphalan, bortezomib, and prednisone^7^. In addition, lenalidomide in combination with dexamethasone or with melphalan and prednisone is uncommonly (0.1–0.99%) associated with arrhythmia but commonly (1.0–9.9% of patients) associated with grade 3–4 tachycardia^67^. Importantly, dexamethasone itself is uncommonly associated with myocardial ischemia^105^.

Proteasome inhibitor treatment can also increase the risk of cardiac complications. A pooled analysis of safety data from European SmPCs showed that cardiac failure is a common ADR associated with carfilzomib^55^ and an uncommon ADR in patients treated with bortezomib^50^. Rates of cardiac failure of grade 3 or above in patients treated with carfilzomib-based regimens in phase 3 trials were in the range 2–4.3%^16,18,32^, and cardiac AEs at grade 3 or higher affected 0–7.5% of those who received bortezomib during phase 3 clinical trials^12,19,21–23,30^. In the ENDEAVOR trial, which directly compared carfilzomib plus dexamethasone with bortezomib plus dexamethasone, the rates of cardiac failure at grade 3 or higher were 2.8 and 0.7%, respectively^18^. However, an ENDEAVOR cardiac substudy of 159 patients (80 receiving carfilzomib and 79 bortezomib) assessing change from baseline LVEF found that six patients (three receiving carfilzomib and three bortezomib) had a significant decrease in LVEF at any time during the study. Furthermore, at > 3 years of follow-up, there was no objective evidence of subclinical or clinically relevant decline in cardiac function (defined as LVEF, right ventricular function, and pulmonary artery systolic pressure) for carfilzomib versus bortezomib. Importantly, reported reductions in LVEF with either agent were mostly reversible^102^.

General clinical guidelines on the diagnosis and treatment of chronic and acute heart failure are published by several bodies including the ESC^106^. ACE inhibitors and β-blockers are the recommended first-line treatment for symptomatic heart failure with reduced ejection fraction; if patients remain symptomatic with an LVEF of 35% or below, a mineralocorticoid receptor antagonist may be added. Other treatment options for selected patients include angiotensin receptor-neprilysin inhibitors and ivabradine. An implantable cardioverter defibrillator and cardiac resynchronization therapy may be indicated if a patient’s LVEF remains at or below 35% despite optimal medical treatment. Diuretics may be used to relieve the symptoms of fluid retention^106^.

As previously discussed, patients with MM can develop cardiac amyloidosis. Such patients may present with dyspnea (particularly when lying down), edema, and angina when exercising^107^. Clinical investigations are likely to show common features of amyloidosis such as congestive heart failure with angiographically normal coronaries, an electrocardiogram with low voltage, bradycardia, arterioventricular block, arrhythmia with conduction delays, wall thickening and/or diastolic dysfunction, elevated serum troponins, and elevated levels of BNP or NTpro-BNP^34^.

Treatment with anti-MM therapy or autologous SCT can be used to reduce the quantity of immunoglobulin secreted into the blood and therefore prevent further deposition in cardiac tissue^108^. Cardiac failure caused by AL amyloidosis can be treated with diuretics but ACE inhibitors and angiotensin II inhibitors are poorly tolerated by patients with cardiac amyloidosis, and the use of such agents can cause profound hypotension^109^.

The ESC recommends assessment of cardiac function before anthracycline treatment is initiated and discussion with the cardiology team if systolic dysfunction or significant valvular heart disease is found^64^. A second assessment should be performed at the end of treatment, especially when the patient has risk factors for cardiac toxicity or if further treatment with potentially cardiotoxic agents is planned. A further assessment should be considered after a cumulative doxorubicin dose of 240 mg/m^2^ for patients with high baseline risk or minor echocardiographic abnormalities^64^.

For patients receiving carfilzomib, additional guidance on reducing risk and fluid monitoring is given in the European SmPC^55^. All patients should be monitored for evidence of volume overload; the total volume of fluids may be adjusted as clinically indicated in patients with baseline cardiac failure or in those at risk of cardiac failure. Individuals with signs or symptoms of New York Heart Association (NYHA) class III or IV cardiac failure, those who have had a recent myocardial infarction, and those with uncontrolled angina or arrhythmias should have a comprehensive medical assessment before starting carfilzomib. Treatment with carfilzomib should be stopped if grade 3 or 4 cardiac events occur; the decision on whether treatment can be restarted at a reduced dose can be taken once the events have been resolved^55^.

The American College of Cardiologists recommends that a cardiologist is consulted before carfilzomib treatment is initiated in patients with a history of heart failure, arrhythmia, pulmonary hypertension, or poorly controlled systemic hypertension^110^.

### Hypertension

In a study of patients newly diagnosed with MM, 38% had a history of hypertension^111^, and data from an American study of patients who had received corticosteroids and at least three other antimyeloma treatments indicated that 36% of participants had hypertension^4^.

Hypertension is relatively common in patients treated with proteasome inhibitors. In a pooled analysis of safety data from clinical trials and postmarketing studies, hypertension was listed as a common ADR for bortezomib^50^ and a very common ADR for carfilzomib^55^. During phase 3 trials, the incidence of grade 3 or 4 hypertension was in the range 0–4% for bortezomib-based regimens^24,25,31^, and 3–15% for carfilzomib-based treatments^16–18,24,32^. In the ENDEAVOR trial the incidence of grade 3 or higher hypertension was 15% in the carfilzomib arm and 3% in the bortezomib arm^18^.

Treatment with the monoclonal antibody daratumumab is also associated with hypertension. Grade 3 hypertension occurred in 5% of patients treated with daratumumab according to a pooled analysis of three open-label clinical trials, and was also reported in 6.6% of patients who received daratumumab in combination with bortezomib and low-dose dexamethasone during the CASTOR phase 3 trial (vs 0.8% in the bortezomib and low-dose dexamethasone arm)^25,112^. However, in the ELOQUENT-2 trial, grade 3 or 4 hypertension was reported in 1.3% patients receiving elotuzumab in combination with lenalidomide/dexamethasone, compared with 2.2% of those treated with lenalidomide/dexamethasone alone^33^.

Several bodies have published recommendations for the treatment of hypertension. The Joint National Committee on the Prevention, Detection, Evaluation and Treatment of High Blood Pressure recommends thiazide-type diuretics for the treatment of stage 1 hypertension (systolic blood pressure 140–159 mmHg and diastolic blood pressure 90–99 mmHg) without compelling comorbidities^113^. Individuals with stage 2 hypertension (systolic blood pressure ≥ 160 mmHg and diastolic blood pressure ≥ 100 mmHg) should be prescribed a thiazide-type diuretic combined with an ACE inhibitor or an angiotensin receptor blocker or a β-blocker. Specific guidelines are given for patients with hypertension and heart failure. ACE inhibitors are recommended for patients with hypertension and stage A (NYHA class I) heart failure. The combination of an ACE inhibitor and a β-blocker is recommended for patients with hypertension and stage B or C (NYHA class II or III) heart failure^113^.

For patients receiving carfilzomib, specific guidance on managing hypertension is available in the European SmPC^55^. Patients’ blood pressure should be monitored before starting treatment with carfilzomib, and particular attention should be given to those with a history of hypertension to ensure that it remains well controlled. Blood pressure should be monitored during treatment, and patients should be assessed for fluid overload before and after each administration of carfilzomib. If hypertension cannot be controlled, the carfilzomib dose should be reduced. When the patient’s blood pressure returns to the baseline level, the benefit–risk ratio of resuming treatment with carfilzomib should be assessed on an individual basis^55^.

### QTc prolongation

Cardiac events at any grade (most frequently atrial fibrillation, tachycardia, palpitation, and sinus tachycardia) were reported in 17.6% of patients who received panobinostat at a dose of 20 mg (in combination with bortezomib and dexamethasone) during the phase 3 PANORAMA-1 trial^88^. Panobinostat, however, can also prolong cardiac ventricular repolarization (corrected QT interval; QTc). Pooled data from patients who received single-agent panobinostat for multiple indications showed that at least 5% of individuals treated at doses of 60 mg or higher had grade 3 QTc prolongation ( > 500 ms without clinical arrhythmia); however, the risk of QTc prolongation does not appear to increase over time, and the recommended dose for patients with MM is 20 mg^88^. No QTc prolongation above 500 ms or episodes of torsades de pointes were observed in the PANORAMA-1 study; however, it should be noted that patients with impaired cardiac function, QTc > 450 ms, or other clinically significant heart or vascular disease (e.g., uncontrolled hypertension) were not eligible for the trial^26^. The European SmPC states that electrocardiograms should be obtained and electrolytes should be assessed prior to initiating treatment with panobinostat and periodically during treatment, particularly in patients with severe gastrointestinal adverse reactions^88^.

## Role of the cardiologist

In the majority of cases, the initial assessment of a patient’s cardiac status, monitoring of cardiac function during treatment, and administration of primary preventative therapies will be carried out by the hemato-oncologist. Similarly, in most centers, the management of uncomplicated cardiovascular AEs during treatment is also undertaken by the hemato-oncologist. A cardiologist may be consulted before initiating treatment for MM if the patient has a history of heart disease, uncontrolled hypertension, left ventricular dysfunction, or symptomatic heart failure, or if abnormal results are observed on the electrocardiogram, echocardiogram, or multigated acquisition scan. A cardiologist may also be consulted during treatment if patients develop any of these conditions. In addition, cardiologists have an important role to play in the education and training of hemato-oncologists on the diagnosis and management of cancer-related and cancer-therapy-related cardiovascular events. The need for close collaboration between these specialties is emphasized by published commentary on the potential benefits of focusing on cardio-oncology in MM^114^.

## Conclusions

The management of cardiovascular risk factors and AEs is an emerging area of interest among the MM community. Maintaining cardiovascular health has become more clinically relevant as the number of treatments and the life expectancy of patients with MM have increased. Three broad factors contribute to the overall cardiovascular risk in this patient population: age-related comorbidities, disease-related risk factors, and increased risk from treatments associated with cardiovascular toxicity. It may not always be clear which of these factors, or indeed which combination of factors, is the underlying cause of any observed cardiovascular AE.

The emergence of several new agents and new classes of agent for the treatment of patients with MM has dramatically improved survival outcomes, placing even more importance on the maintenance of cardiac function. Several of the new agents are associated with cardiovascular toxicity and are routinely used in three-drug combinations with the potential for additive cardiovascular risk. However, with careful risk assessment, monitoring, and prophylactic treatment, many of the cardiovascular AEs associated with MM treatments can be prevented or identified and managed at an early stage, which may limit the risk of long-term cardiac impairment. In order to extend these treatments to all patients who would benefit from them, it is important that perceived cardiovascular risk does not form an inappropriate barrier to treatment.

A multidisciplinary approach involving hemato-oncologists and cardiologists can help to optimize outcomes and expand access to new treatments for patients with MM and cardiac comorbidities. In addition to providing clinical guidance, cardiologists have a role to play in educating and training other members of the healthcare team involved in the diagnosis and treatment of the cardiac AEs that patients with MM may experience during treatment.

It is important to recognize that, in general, oncology trials have not been powered to detect all cardiovascular events or to compare the differences between treatment arms systematically. The current clinical guidelines represent the best available expert opinion. The guidance, however, must be tested in future clinical trials designed to collect data that will increase our understanding of the cardiovascular risks associated with cancer treatments and the optimal management of the resulting AEs. Specific evidence-based guidelines can then be developed to facilitate individualized treatment decisions and optimize outcomes for patients.

## Supplementary information

Supplementary Table 1.

Supplementary Table 2.
