# Supplementary material for: Management of cardiovascular risk in patients with multiple myeloma
Source: Blood Cancer J. 2019 Feb 26;9(3):26. doi: 10.1038/s41408-019-0183-y (PMC6391463; doi:10.1038/s41408-019-0183-y)
Supplement: Supplementary file 1 — Supplementary Table 1. [file 41408_2019_183_MOESM1_ESM.docx]

Supplementary Table 1. Definitions of selected cardiovascular adverse events according to the Common Terminology Criteria for Adverse Events (version 5)^6^.

|  | **Grade** | | | |
| --- | --- | --- | --- | --- |
| **Adverse event** | **1** | **2** | **3** | **4** |
| **Atrial fibrillation** | *A dysrhythmia without discernible P waves and an irregular ventricular response due to multiple reentry circuits. The rhythm disturbance originates above the ventricles* | | | |
|  | Asymptomatic, intervention not indicated | Nonurgent medical intervention indicated | Symptomatic, urgent intervention indicated; device (e.g., pacemaker); ablation; new onset | Life-threatening consequences; embolus requiring urgent intervention |
| **Heart failure** | *The inability of the heart to pump blood at an adequate volume to meet tissue metabolic requirements, or, the ability to do so only at an elevation in the filling pressure* | | | |
|  | Asymptomatic with laboratory (e.g. BNP) or cardiac imaging abnormalities | Symptoms with moderate activity or exertion | Symptoms at rest or with minimal activity or exertion; hospitalization; new onset of symptoms | Life-threatening consequences; urgent intervention indicated (e.g. continuous IV therapy or mechanical hemodynamic support) |
| **Palpitations** | *An unpleasant sensation of irregular and/or forceful beating of the heart* | | | |
|  | Mild symptoms; intervention not indicated | Intervention indicated | *–* | *–* |
| **Myocardial infarction** | *Gross necrosis of the myocardium; this is due to an interruption of blood supply to the area* | | | |
|  | – | Asymptomatic and cardiac enzymes minimally abnormal and no evidence of ischemic ECG changes | Severe symptoms; cardiac enzymes abnormal; hemodynamically stable; ECG changes consistent with infarction | Life-threatening consequences; hemodynamically unstable |
| **Sinus bradycardia** | *A dysrhythmia with a heart rate less than 60 beats per minute that originates in the sinus node* | | | |
|  | Asymptomatic, intervention not indicated | Symptomatic, intervention not indicated; change in medication initiated | Symptomatic, intervention indicated | Life-threatening consequences; urgent intervention indicated |
| **Sinus tachycardia** | *A dysrhythmia with a heart rate greater than 100 beats per minute that originates in the sinus node* | | | |
|  | Asymptomatic, intervention not indicated | Symptomatic; non-urgent medical intervention indicated | Urgent medical intervention indicated | – |
| **Hypertension^a^** | *Pathological increase in blood pressure* | | | |
|  | Systolic BP 120–139 mm Hg or diastolic BP 80–89 mm Hg | Systolic BP 140–159 mm Hg or diastolic BP 90–99 mm Hg if previously WNL; change in baseline medical intervention indicated; recurrent or persistent (≥24 hrs); symptomatic increase by >20 mm Hg (diastolic) or to >140/90 mm Hg; monotherapy indicated initiated | Systolic BP ≥160 mm Hg or diastolic BP ≥100 mm Hg; medical intervention indicated; more than one drug or more intensive therapy than previously used indicated | Life-threatening consequences (e.g., malignant hypertension, transient or permanent neurologic deficit, hypertensive crisis); urgent intervention indicated |
| **Hypotension** | *A blood pressure that is below the normal expected for an individual in a given environment* | | | |
|  | Asymptomatic, intervention not indicated | Non-urgent medical intervention indicated | Medical intervention indicated; hospitalization indicated | Life-threatening consequences and urgent intervention indicated |
| **Thromboembolic event** | *Occlusion of a vessel by a thrombus that has migrated from a distal site via the blood stream* | | | |
|  | Medical intervention not indicated (e.g., superficial thrombosis) | Medical intervention indicated | Urgent medical intervention indicated (e.g., pulmonary embolism or intracardiac thrombus) | Life-threatening consequences with hemodynamic or neurologic instability |

BNP, B-type natriuretic peptide; BP, blood pressure; ECG, electrocardiogram; IV, intravenous; ULN, upper limits of normal; WNL, within normal limits.

1. Definitions shown are for adult patients only.
