# Supplementary material for: Management of cardiovascular risk in patients with multiple myeloma
Source: Blood Cancer J. 2019 Feb 26;9(3):26. doi: 10.1038/s41408-019-0183-y (PMC6391463; doi:10.1038/s41408-019-0183-y)
Supplement: Supplementary file 2 — Supplementary Table 2. [file 41408_2019_183_MOESM2_ESM.docx]

Supplementary Table 2. Cardiovascular adverse events reported in key phase 3 trials involving agents used in the treatment of patients with RRMM.

|  | **Trial**  **name or first author** | **Treatment arms** | **Randomized patients, n** | **Duration**  **(months)** | **Cardiac adverse events** | **Vascular adverse events** |
| --- | --- | --- | --- | --- | --- | --- |
| Anthracyclines | Orlowski^20^ | **PEGylated liposomal doxorubicin** 30 mg/m^2^ IV on day 4 of each 21-day cycle + **bortezomib** 1.3 mg/m^2^ IV on days 1, 4, 8 and 11 | 324 | 7.2 | **PEGylated liposomal doxorubicin + bortezomib vs bortezomib**  *Grade 2 or above, treatment-related*  Congestive heart failure^a^: 2% vs 1%  Arrhythmia^b^: 2% vs 1%  Coronary ischemic disease^c^: 1% vs 0%  *Grade 3 or 4*  Cardiac events: 2% vs 3% | **PEGylated liposomal doxorubicin + bortezomib vs bortezomib**  *Grade 3 or 4*  Thromboembolic events: 1% vs 1% |
|  |  | **Bortezomib** 1.3 mg/m^2^ IV on days 1, 4, 8, and 11 of each 21-day cycle | 322 |  |  |  |
| Proteasome inhibitors | VISTA^22^ | **Bortezomib** 1.3 mg/m^2^ IV on days 1, 4, 8, 11, 22, 25, 29, and 32 of each 6-week cycle for cycles 1–4 and on days 1, 8, 22, and 29 for cycles 5–9 + **melphalan** 9 mg/m^2^ oral on days 1–4 of each 6-week cycle + **prednisone** 60 mg/m^2^ oral on days 1–4 of each 6-week cycle | 344 | 16.3 | **Bortezomib** + **melphalan + prednisone vs melphalan + prednisone**  None reported | **Bortezomib + melphalan + prednisone vs melphalan + prednisone**  *Grade 3*  Deep vein thrombosis: 1% vs 1% |
|  |  | **Melphalan** 9 mg/m^2^ oral on days 1– 4 of each 6-week cycle + **prednisolone** 60 mg/m^2^ on days 1–4 of each 6-week cycle | 338 |  |  |  |
|  | PATHEMA/  GEM (induction)^30^ | **Bortezomib** 1.3 mg/m^2^ IV on days 1, 4, 8, and 11, at 3-week intervals + **thalidomide** 50 mg oral on days 1–14 of cycle 1, 100 mg on days 15–28 of cycle 1 and 200 mg on day 1–28 of cycle 2 + **dexamethasone** 40 mg/day oral on days 1–4 and 9–12 of each cycle | 130 | 35.2 | **Bortezomib + thalidomide + dexamethasone vs thalidomide + dexamethasone vs VBMCP/VBAD/V**  None reported | **Bortezomib + thalidomide + dexamethasone vs thalidomide + dexamethasone vs VBMCP/VBAD/B**  *Grade 3 or 4*  Deep vein thrombosis/pulmonary embolism: 12% vs 5% vs 4% |
|  |  | **Thalidomide** 50 mg oral on day 1 of the first 28-day cycle, 100 mg on day 15 of cycle 1 and 200 mg on day 1 of cycle 2 + **dexamethasone** 40 mg/day oral on days 1–4 and 9–12 of each cycle | 127 |  |  |  |
|  |  | **VBMCP/VBAD/V**  **Vincristine** 0.03 mg/kg IV on day 1 + **carmustine** 0.5 mg/kg IV on day 1 + **cyclophosphamide** 10 mg/kg IV on day 1 + **melphalan** 0.25 mg/kg oral on days 1–4 + **prednisone** 1 mg/kg oral on days 1–4; 0.5 mg/kg on days 5–8 and 0.25 mg/kg on days 9–12  Alternating cycles with **vincristine** 1 mg IV on day 1 + **carmustine** 30 mg/m^2^ IV day 1 + **doxorubicin** 40mg/m^2^ IV on day 1 + **dexamethasone** 40 mg oral on days 1–4, 9–12, and 17 to 20 for a total of 4 cycles followed by **bortezomib** 1.3 mg/m^2^ IV on days 1, 4, 8, and 11 of each 28-day cycle for 2 cycles | 129 |  |  |  |
|  | PATHEMA/GEM (maintenance)^27^ | Following induction as above:  Autologous stem cell transplantation (MEL-200), followed by maintenance with: |  |  |  |  |
|  |  | **Alfa2-IFN** (starting dose of 1.5 MU subcutaneously  three times per week; could be increased to 3 MU at investigator discretion depending on the tolerance) | 92 |  | **Alfa-IFN vs thalidomide vs thalidomide + bortezomib**  None reported | **Alfa-IFN vs thalidomide vs thalidomide + bortezomib**  None reported |
|  |  | **Thalidomide** 100 mg per day orally | 88 |  |  |  |
|  |  | **Thalidomide** 100 mg per day orally plus  one cycle of IV **bortezomib** on days 1, 4, 8, and 11 every 3 months | 91 |  |  |  |
|  | IFM2005-01^19^ | **Bortezomib** 1.3 mg/m^2^ IV on days 1, 4, 8, and 11 of each 21-day cycle for 4 cycles + **dexamethasone** 40 mg/day orally on days 1–4 of all cycles and on days 9–12 of cycles 1 and 2 | 240 | 32.2 | **Bortezomib** + **dexamethasone vs vincristine** + **doxorubicin** + **dexamethasone**  *Grade 1–4*  Cardiac disorders: 5.9% vs 5.9% | **Bortezomib** + **dexamethasone vs vincristine** + **doxorubicin** + **dexamethasone**  *Grade 3 or 4*  Thrombosis: 1.7% vs 5.4% |
|  |  | **Vincristine** 0.4 mg/day IV on days 1–4 of each 4-week cycle + **doxorubicin** 9 mg/m^2^ IV on days 1–4 of each 4 week cycle + **dexamethasone** 40 mg orally on days 1–4 of all cycles and on days 9–12 and days 17–20 of cycles 1 and 2 | 242 |  |  |  |
|  | MMVAR/IFM 2005-04^12^ | **Bortezomib** 1.3 mg/m^2^ IV on days 1, 4, 8, and 11 of each 21-day cycle for 8 cycles, then same dose on days 1, 8, 15, and 22 of each 42-day cycle for 6 cycles + **thalidomide** 200 mg oral every day for 1 year + **dexamethasone** 40 mg/day oral on days 1–4 of each 21-day cycle for 1 year | 135 | 30 | **Bortezomib** + **thalidomide** + **dexamethasone vs thalidomide** + **dexamethasone**  *Grade 3 or 4*  Cardiac adverse events: 2% vs 1% | **Bortezomib** + **thalidomide** + **dexamethasone vs thalidomide** + **dexamethasone**  *Grade 3 or 4*  Thromboembolism: 6% vs 5% |
|  |  | **Thalidomide** 200 mg/day oral every day for 1 year + **dexamethasone** 40 mg/day oral on days 1–4 of each 21-day cycle for 1 year | 134 |  |  |  |
|  | APEX^21^ | **Bortezomib** 1.3 mg/m^2^ IV on days 1, 4, 8, and 11 of each 21-day cycle for cycles 1–8 then on days 1, 8, 15, and 22 of each 35-day cycle for cycles 9–11 | 333 | 8.3 | **Bortezomib vs dexamethasone**  *All grades*  Cardiac disorders: 15% vs 13%  Congestive cardiac failure: 2% vs 2% | **Bortezomib vs dexamethasone**  None reported |
|  |  | **Dexamethasone** 40 mg oral on days 1–4, 9–12, and 17–20 of each 35-day cycle for cycles 1­–4, then days 1–4 of each 28-day cycle for cycles 5–9 | 336 |  |  |  |
|  | SWOG SO777^23^ | **Bortezomib** 1.3 mg/m^2^ IV on days 1, 4, 8, and 11 of each 21-day cycle + **lenalidomide** 25 mg oral on days 1–14 + **dexamethasone** 20 mg oral on days 1, 2, 4, 5, 8, 9, 11, and 12 for 8 cycles | 264 | 54 | **Bortezomib** + **lenalidomide** + **dexamethasone vs lenalidomide** + **dexamethasone**  *Grade 3*  General cardiac toxic events: 7.5% vs 3.5% | **Bortezomib** + **lenalidomide** + **dexamethasone vs lenalidomide** + **dexamethasone**    *Grade 3 or 4*  Vascular toxic events: 9.1% vs 9.3% |
|  |  | **Lenalidomide** 25 mg oral on days 1–21 of each 28-day cycle + **dexamethasone** 40 mg oral on days 1, 8, 15, and 22 for 6 cycles | 261 | 56 |  |  |
|  | Kropff^31^ | **Bortezomib** 1.3 mg/m^2^ IV on days 1, 4, 8, and 11 of each 21-day cycle + **dexamethasone** 20 mg oral on days 1, 2, 4, 5, 8, 9, 11, and 12 | 48 | 5 cycles | **Bortezomib** + **dexamethasone vs bortezomib** + **dexamethasone + cyclophosphamide**  *Grade 3 or above* in ≥ 4% patients  None reported | **Bortezomib** + **dexamethasone vs bortezomib** + **dexamethasone + cyclophosphamide**  Grade 3 or above in ≥ 4% patients  Hypertension: 4.3% vs 0.0% |
|  | TOURMALINE-MM1^13, 14^ | **Ixazomib** 4 mg/day oral on days 1, 8, and 15 of each 28-day cycle + **lenalidomide** 25 mg/day oral on days 1–21 + **dexamethasone** 40 mg/day oral on days 1, 8, 15, and 22 | 360 | 23 | **Ixazomib** + **lenalidomide** + **dexamethasone vs placebo** + **lenalidomide** + **dexamethasone**  *Grade 3 or 4*  Arrhythmia: 5.9% vs 3.1%  Heart failure: 2.5% vs 1.7%  Myocardial infarction: 0.8% vs 1.1%  Subgroup analysis by cytogenetic risk group  High-risk (n = 136)  *Grade 3 or above*  Arrhythmia: 5.4% vs 3.2%  Heart failure: 1.4% vs 1.6%  Standard-risk (n = 414)  *Grade 3 or above*  Arrhythmia: 6.5% vs 5.1%  Heart failure: 4.0% vs 3.3% | **Ixazomib** + **lenalidomide** + **dexamethasone vs placebo** + **lenalidomide** + **dexamethasone**  *Grade 3 or 4*  Hypertension: 3.0% vs 1.0%  Thromboembolism: 3.0% vs 3.3%  Hypotension: 1.0% vs 0.3%  Subgroup analysis by cytogenetic risk group  High-risk (n = 136)  *Grade 3 or above*  Venous embolic and thrombotic events: 4.1% vs 4.8%  Standard-risk (n = 414)  *Grade 3 or above*  Venous embolic and thrombotic events: 3.0% vs 3.3% |
|  |  | **Placebo (for ixazomib)** + **lenalidomide** 25 mg oral on days 1–21 of each 28-day cycle + **dexamethasone** 40 mg/day oral on days 1, 8, 15, and 22 | 362 |  |  |  |
|  | ASPIRE^17, 32^ | **Carfilzomib** 20 mg/m^2^ IV on days 1 and 2, and 27 mg/m^2^ on days 8, 9, 15, and 16 of first 28-day cycle and 27 mg/m^2^ thereafter for cycles 2–12, then 27 mg/m^2^ on days 1, 2, 15, and 16 of cycles 13–18 + **lenalidomide** 25 mg oral on days 1–21 + **dexamethasone** 40 mg oral on days 1, 8, 15, and 22 | 396 | 67.1 | **Carfilzomib + lenalidomide + dexamethasone vs lenalidomide + dexamethasone**  *Grade 3 or above*  Cardiac failure^d^: 4.3% vs 2.1%  Ischemic heart disease^e^: 3.8% vs 2.3%  Fatal treatment-emergent cardiac disorders: 2.6% vs 2.3% | **Carfilzomib + lenalidomide + dexamethasone vs lenalidomide** + **dexamethasone**  *Grade 3 or above*  Hypertension: 6.4% vs 2.3%  Interim analysis (follow up 32.3 and 31.5 months, respectively) reported:  Pulmonary embolism: 3.1% vs 2.3%  Deep vein thrombosis: 1.8% vs 1.0% |
|  |  | **Lenalidomide** 25 mg oral on days 1–21 of each 28-day cycle + **dexamethasone** 40 mg oral on days 1, 8, 15, and 22 | 396 |  |  |  |
|  | ENDEAVOR^18, 24^ | **Carfilzomib** 20 mg/m^2^ IV on days 1 and 2 then 56 mg/m^2^ on days 8, 9, 15, and 16 of first 28-day cycle, 56 mg/m^2^ on days 1, 2, 8, 9, 15, and 16 of all cycles + **dexamethasone** 20 mg oral/IV on days 1, 2, 8, 9, 15, 16, 22, and 23 | 464 | 37.5 | **Carfilzomib** + **dexamethasone vs bortezomib** + **dexamethasone**  *Grade 3 or above*  Cardiac failure: 2.8% vs 0.7%  Ischemic heart disease^f^: 2.6% vs 1.5% | **Carfilzomib** + **dexamethasone vs bortezomib** + **dexamethasone**  *Grade 3 or above*  Hypertension: 14.5% vs 3.3%  Deep vein thrombosis: 0.9% vs 0.7%  Pulmonary embolism: 1.9% vs 0.9% |
|  |  | **Bortezomib** 1.3 mg/m^2^ IV/SC on days 1, 4, 8, and 11 of each 21-day cycle + **dexamethasone** 20 mg oral/IV on days 1, 2, 4, 5, 8, 9, 11, and 12 of each 21-day cycle | 465 | 36.9 |  |  |
|  | FOCUS^16^ | **Carfilzomib** 20 mg/m^2^ IV on days 1 and 2, and 27 mg/m^2^ on days 8, 9,15, and 16 of the first 28-day cycle, then 27 mg/m^2^ on days 1, 2, 8, 9, 15, and 16 of cycles 2–9 | 157 | 27.8 | **Carfilzomib vs corticosteroids** + **optional cyclophosphamide**  *Grade 3 or above*  Cardiac failure: 2% vs 1% | **Carfilzomib vs corticosteroids** + **optional cyclophosphamide**  *Grade 3 or above*  Hypertension: 3% vs 0% |
|  |  | **Prednisone** 30 mg/day oral or **dexamethasone** 6 mg every other day + **cyclophosphamide** 50 mg/day oral to maximum of 1400 mg/cycle (optional) | 158 | 29.8 |  |  |
| Immunomodulators | Dimopoulos^9^ | **Lenalidomide** 25 mg/day oral on days 1–21 of each 28‑day cycle + **dexamethasone** 40 mg/day oral on days 1–4, 9–12, and 17–20 for cycles 1–4, then days 1–4 thereafter | 176 | 16.4 | **Lenalidomide** + **dexamethasone vs placebo** + **dexamethasone**  None reported | **Lenalidomide** + **dexamethasone vs placebo** + **dexamethasone**  *Grade 3*  Deep vein thrombosis: 3.4% vs 2.9%  Pulmonary embolism: 1.1% vs 0.6%  VTE: 7.4% vs 3.5%  *Grade 4*  Deep vein thrombosis: 0.6% vs 0.6%  Pulmonary embolism: 3.4% vs 0.6%  VTE: 4.0% vs 1.1% |
|  |  | **Placebo (for lenalidomide)** + **dexamethasone** 40 mg/day oral on days 1–4, 9–12, and 17–20 of cycles 1–4, then days 1–4 thereafter | 175 |  |  |  |
|  | Weber^10^ | **Lenalidomide** 25 mg/day oral on days 1–21 of each 28‑day cycle + **dexamethasone** 40 mg/day oral on days 1–4, 9–12, and 17–20 for cycles 1–4, then days 1–4 thereafter | 177 | 17.6 | **Lenalidomide** + **dexamethasone vs placebo** + **dexamethasone**  None reported | **Lenalidomide** + **dexamethasone vs placebo** + **dexamethasone**  *Grade 3*  Deep vein thrombosis: 11.9% vs 3.4%  Pulmonary embolism: 0.6% vs 0%  VTE: 11.9% vs 2.9%  *Grade 4*  Deep vein thrombosis: 0% vs 0%  Pulmonary embolism: 2.8% vs 0.6%  VTE: 2.8% vs 0.6% |
|  |  | **Placebo (for lenalidomide)** + **dexamethasone** 40 mg/day oral on days 1–4, 9–12, and 17–20 of each 28-day cycle for cycles 1–4, then days 1–4 thereafter | 176 | 17.6 |  |  |
|  | Rajkumar^11^ | **Thalidomide** 200 mg oral for 4 weeks + **dexamethasone** 40 mg oral on days 1–4, 9–12, 17–20 of each 28-day cycle | 103 | 4 cycles | **Thalidomide** + **dexamethasone vs dexamethasone**  *Grade 3 or above*  Sinus bradycardia: 1% vs 0% | **Thalidomide** + **dexamethasone vs dexamethasone**  *Grade 3 or above*  Thrombosis/embolism: 20% vs 3%  Deep vein thrombosis: 17% vs 3%  Hypotension: 4% vs 3%  Hypertension: 0% vs 3% |
|  |  | **Dexamethasone** 40 mg oral on days 1–4, 9–12, 17–20 of each 28-day cycle | 104 |  |  |  |
|  | GEM2005^7^ | **Bortezomib** 1.3 mg/m^2^ IV on days 1, 4, 8, 11, 22, 25, 29, and 32 of each 42-day cycle + **melphalan** 9 mg/m^2^ oral on days 1­–4 + **prednisone** 60 mg/m^2^ oral on days 1–4 for cycle 1 then **bortezomib** 1.3 mg/m^2^ IV on days 1, 8, 15, and 22 + **melphalan** 9 mg/m^2^ oral on days 1­–4 + **prednisone** 60 mg/m^2^ oral on days 1–4 for cycles 2–6 | 130 | 6 cycles | **Bortezomib** + **melphalan** + **prednisone vs bortezomib** + **thalidomide**+ **prednisone**  *Grade 3 or above*  Cardiac events:0% vs 8% | **Bortezomib** + **melphalan** + **prednisone** vs **bortezomib** + **thalidomide**+ **prednisone**  *Grade 3 or above*  Deep vein thrombosis/thromboembolism: <1% vs 2% |
|  |  | **Bortezomib** 1.3 mg/m^2^ IV on days 1,4, 8, 11, 22, 25, 29, and 32 of each 42-day cycle + **thalidomide** 100 mg oral on days 1­–42 + **prednisone** 60 mg/m^2^ oral on days 1–4 for cycle 1 then **bortezomib** 1.3 mg/m^2^ IV on days 1, 8, 15, and 22 of each 35-day cycle + **thalidomide** 100 mg oral on days 1­–34 + **prednisone** 60 mg/m^2^ oral on days 1–4 cycles 2–6. | 130 |  |  |  |
|  | MM-003^15^ | **Pomalidomide** 4 mg oral on days 1–21 of each 28-day cycle for cycle 1 + **dexamethasone** 40 mg oral on days 1, 8, 15 and 22 | 302 | 10 | **Pomalidomide** + **dexamethasone vs dexamethasone**  None reported | **Pomalidomide** + **dexamethasone vs dexamethasone**  *Grade 3 or 4*  Deep vein thrombosis or pulmonary embolism  1% vs 0% |
|  |  | **Dexamethasone** 40 mg oral on day 1–4, 9–12 and 17–20 of each 28-day cycle | 153 |  |  |  |
| Monoclonal antibodies | ELOQUENT-2^8, 28, 33^ | **Elotuzumab** 10 mg/kg IV on days 1, 8, 15, and 22 of each 28-day cycle for cycles 1 and 2, and days 1 and 15 thereafter + **lenalidomide** 25 mg/day oral on days 1–21 + **dexamethasone** 40 mg oral in weeks without elotuzumab and 8 mg IV and 28 mg oral on the day elotuzumab was administered | 321 | 33 | **Elotuzumab** + **lenalidomide** + **dexamethasone vs lenalidomide** + **dexamethasone**  *Grade 3 or 4*  Cardiac failure: 0.3% vs 0.6% | **Elotuzumab** + **lenalidomide** + **dexamethasone vs lenalidomide** + **dexamethasone**  *Grade 3 or 4*  Deep vein thrombosis: 6.3% vs 2.5%  Hypertension: 1.3% vs 2.2% |
|  |  | **Lenalidomide** 25 mg oral on days 1–21 of each 28-day cycle + **dexamethasone** 40 mg oral on days 1, 8, 15, and 22 | 325 |  |  |  |
|  | CASTOR^25^ | **Daratumumab** 15 mg/kg IV on days 1, 8, and 15 of each 21–day cycle for cycles 1–3, day 1 for cycles 4–8 and every 4 weeks thereafter + **bortezomib** 1.3 mg/m^2^ SC on days 1, 4, 8, and 11 of each 21-day cycle for cycles 1–8 + **dexamethasone** 20 mg oral/IV on days 1, 2, 4, 5, 8, 9, 11, and 12 of each 21-day cycle for cycles 1–8 | 251 | 7.4 | **Daratumumab** + **bortezomib** + **dexamethasone vs bortezomib** + **dexamethasone**  None reported | **Daratumumab** + **bortezomib** + **dexamethasone vs bortezomib** + **dexamethasone**  *Grade 3 or 4*  Hypertension: 6.6% vs 0.8% |
|  |  | **Bortezomib** 1.3 mg/m^2^ SC on days 1, 4, 8, and 11 of each 21-day cycle for cycles1–8 + **dexamethasone** 20 mg/day oral/IV on days 1, 2, 4, 5, 8, 9, 11, and 12 of each 21-day cycle for 1–8 cycles | 247 |  |  |  |
|  | POLLUX^29^ | **Daratumumab**: 16 mg/kg IV on days 1, 8, 15, and 22 of each 56-day cycle for cycles 1–2, then on day 1 and 15 for cycles 3–6 and every 4 weeks thereafter + **lenalidomide** 25 mg oral on days 1–21 of each cycle + **dexamethasone** 40 mg oral weekly | 286 | 13.5 | **Daratumumab** + **lenalidomide** + **dexamethasone vs lenalidomide** + **dexamethasone**  None reported | **Daratumumab** + **lenalidomide** + **dexamethasone vs lenalidomide** + **dexamethasone**  *All grades*  Deep vein thrombosis: 1.8% vs 3.9% |
|  |  | **Lenalidomide** 25 mg oral on days 1–21 of each cycle + **dexamethasone** 40 mg oral weekly | 283 |  |  |  |
| Histone deacetylase inhibitors | PANORAMA^26^ | **Panobinostat** 20 mg/day oral on days 1, 3, 5, 8, 10, and 12 of each 21-day cycle + **bortezomib** 1.3 mg/m^2^ on days 1, 4, 8, and 11 + **dexamethasone** 20 mg/day oral on days 1, 2, 4, 5, 8, 9, 11, and 12 | 387 | 6.5 | **Panobinostat** + **bortezomib** + **dexamethasone vs placebo** + **bortezomib** + **dexamethasone**  None reported | **Panobinostat** + **bortezomib** + **dexamethasone vs placebo** + **bortezomib** + **dexamethasone**  *Grade 3 or 4*  Hypotension: 2.9% vs 1.3% |
|  |  | **Placebo (for** **panobinostat)** + **bortezomib** 1.3 mg/m^2^ IV on days 1, 4, 8, and 11 + **dexamethasone** 20 mg/day oral on days 1, 2, 4, 5, 8, 9, 11, and 12 | 381 | 5.6 |  |  |

IV, intravenous; MedDRA, Medical Dictionary for Regulatory Activities; RRMM, relapsed and/or refractory multiple myeloma; SC, subcutaneous; VBAD, vincristine + carmustine + doxorubicin + dexamethasone; VBMCP, vincristine + carmustine + melphalan + cyclophosphamide + prednisone.

Data shown reflect most recent published values associated with each study; earlier publications are cited for reference.

1. Includes the following MedDRA-derived terms: ventricular dysfunction, cardiac failure, right ventricular failure, congestive cardiac failure, chronic cardiac failure, acute pulmonary edema, and pulmonary edema.
2. Includes the following MedDRA-derived terms: arrhythmia, nodal arrhythmia, palpitation, bradycardia, tachycardia, tachyarrhythmia, sinus bradycardia, sinus tachycardia, atrioventricular block, supraventricular tachycardia, atrial fibrillation, atrial flutter, left bundle branch block, QRS axis abnormal, and extrasystoles.
3. Includes the following MedDRA-derived terms: angina pectoris, myocardial ischemia, and myocardial infraction.
4. Includes: congestive cardiac failure, pulmonary edema, hepatic congestion, cardiopulmonary failure, acute pulmonary edema, acute cardiac failure, and right ventricular failure.
5. Includes: (in descending order of frequency) angina pectoris, myocardial infarction, acute myocardial infarction, increased blood creatinine phosphokinase, coronary artery disease, myocardial ischemia, coronary artery occlusion, increased troponin, increased troponin T, acute coronary syndrome, abnormal cardiac stress test, cardiomyopathy stress, unstable angina, coronary artery stenosis, abnormal electrocardiogram ST-T segment, and abnormal electrocardiogram T wave.
6. Grouped term standardized MedDRA Query narrow.
